# Communicative health literacy in patients with non-communicable diseases in Ethiopia: a cross-sectional study

**DOI:** 10.1186/s41182-021-00345-9

**Published:** 2021-07-13

**Authors:** Desalew Tilahun, Abebe Abera, Gugsa Nemera

**Affiliations:** School of Nursing, Faculty of Health Sciences, Institute of Health, Jimma University, Jimma, Ethiopia

**Keywords:** Communicative heath literacy, Non-communicable diseases, Health literacy questionnaire, Ethiopia

## Abstract

**Background:**

Health literacy plays a prominent role in empowering individuals for prevention as well as management of non-communicable diseases (NCDs). However, there is paucity of information on the health literacy of patients with non-communicable diseases in Ethiopia. Therefore, this study aimed to assess communicative health literacy and associated factors in patients with NCDs on follow-up at Jimma Medical Center (JMC), Ethiopia.

**Methods:**

A cross-sectional study was conducted from 4 May 2020 to 4 July 2020 with 408 randomly selected adult patients, attending outpatient department of JMC in Ethiopia. The final sample size was obtained by using single population proportion formula. All patients with NCDs who were on follow-up at chronic illness clinic, JMC, were used as a source population. All eligible patients with NCDs who fulfilled the inclusion criteria were included in this study. A simple random sampling technique was used to recruit study participants. Data were collected through structured interviewer administered questionnaires on the six of nine health literacy domains using Health Literacy Questionnaire (HLQ) containing 30 items, socio-demographic and socio-economic characteristics, disease-related factors, and health information sources. Multivariable logistic regression was executed to determine the associations.

**Result:**

Descriptive analysis shows more than half of the respondents in four of the six health literacy domains had high communicative health literacy level (CHLL). The proportion of people with high CHLL across each of the domains was as follows: health care provider support (56.1%), social support for health (53.7%), active engagement with a healthcare provider (56.1%), and navigating healthcare system (53.4%). We found educational status was significantly associated with five of six health literacy domains whereas number of sources was associated with four of six health literacy domains.

**Conclusion:**

The overall findings of the current study indicate that health literacy levels vary according to socio-demographic and disease characteristics of patients. Thus, healthcare professionals should assess patients’ health literacy level and tailor information and support to the health literacy skills and personal context of their patients.

## Introduction

Each year, more than one-third of a million people in the world die due to NCDs (Diabetes, cardiovascular diseases, cancers, and chronic respiratory diseases). Of these deaths, approximately 15 million are untimely age 30 through 70 [1] including Ethiopia [2]. The negative effects of the NCD pandemic has been most severe in countries with weak economies and social structures that are struggling to build their health systems to respond to this critical development challenge [1].

The probability of dying prematurely from NCDs is four-fold higher for people living in low- and middle-income countries (LMIC) than in high-income countries [1]. Dramatically, most premature deaths from NCDs representing more than one-fourth of all global deaths could have been prevented [1] through improved health literacy that enables individuals to take an active role in their health [3]. Health literacy (HL) is a fundamental in effective management of NCDs through effective disease prevention systems, and also for the management of NCDs as people must access, understand, and engage in lifelong disease management process [1]. Hence, HL is considered as a key mechanism to improve NCD prevention and management systems [1] and also it shapes people’s health and the safety and quality of health care [4].

Health literacy (HL) represents individuals’ cognitive and social skills to gain access to understand and use information in ways that promote and maintain good health [5]. Communicative/interactive health literacy (CHL) is one of the elements of HL representing more advanced cognitive and literacy skills together with social skills to participate in everyday activities, to extract information and derive meaning from different forms of communication, and to apply new information to the changing circumstances [4]. CHL consists of the health care provider support, social support for health, active engagement with healthcare providers, navigating the healthcare system, ability to find good quality information, and actively managing my health [6] health literacy domains.

Understanding the health literacy level (HLL) is foundational compliance to treatment and improved use of health care services in patients with NCDs [7]. The HLL of patients with NCDs can positively or negatively affect their health outcomes [8]. Patients with low HLL often have increased complications of diseases [9], high rates of comorbidities [10], high health expenditure [11], poor physical functioning [12], lower receipt of preventive healthcare services, suboptimal medications adherence [13], higher mortality rates, higher rates of hospitalization [13, 14], difficulty in counseling [15], dearth of skills to easily access health information, less likely to engage in active discussions about their health options [16], difficulty in comprehending health care information [10], experience misunderstanding about their disease, and communicate ineffectively with healthcare providers [17].

On the other hand, high health literacy level (high HLL) is a critical component to lower risk of developing NCDs [1]; may reduce disease impact [12]; enables chronic patients to take an active role in their health [3]; is a milestone for clients in chronic diseases to communicate with health professionals to describe their needs [16]; can also support a better NCDs management, including changed personal behaviors, social actions for health, and the capability of influencing others towards healthy decisions [11]; and manage long-term conditions effectively [16]. Evidence shows that health literacy level (HLL) varies between countries and between domains. Given that in Slovak high HLL was seen for three of nine HL domains: social support for health (70%); ability to find good health information (70%); and understanding health information well enough to know what to do (75.6%) [18]. In Nepal, high HLL was seen for HL domains such as healthcare provider support (21%), having sufficient information (23.5%), social support for health (22.7%), ability to find the good health information (24.8%), and reading and understanding the health information (25.2%) [19].

Several studies [3, 7, 20–25] indicated that variances in CHLL across the world within health literacy domains are linked either positively or negatively with socio-demographic and socio-economic characteristics, health information sources (watching medical-related television series, health professionals, family members, friends, radio, Internet, books), and disease-related factors (number of chronic diseases, complications from chronic diseases, and follow-up visit last 4 weeks at clinic) which were associated with HL. Despite the fact that health literacy is a pre-requisite for better health outcomes in patients with chronic diseases [26], it has received little attention in the literature on NCDs, in Ethiopia in this population. To the best of researcher’s knowledge and search level using different databases, there is no study on health literacy in Ethiopia. Thus, the aim of this study was to describe CHL and associated factors in patients with NCDs on follow-up at JMC.

## Methods

### Study design, setting, period, and population

A cross-sectional study was performed at a chronic illness clinic. JMC is one of the oldest public hospitals that provides inpatient, outpatient, and emergency and chronic clinic follow-up service for an estimated 15 million people in the southwest part of the country. Approximately, 16,000 patients attend the follow-up clinic for NCDs, in a year. During the month-long data collection from 4 May 2020 to 4 July 2020, there were 1200 patients with cardiovascular diseases (CVD), 150 patients with chronic obstructive pulmonary diseases (COPD), 200 patients with asthma, 1220 patients with diabetes mellitus (DM), 1200 patients with hypertension (HTN), and 1100 patients with epilepsy.

### Study participants, sample size determination, and sampling procedures

Individuals aged 18 years and above who are on follow-up at JMC for abovementioned diseases, were not seriously ill, and had no hearing impairments during data collection period were included in the study. A final sample size of 422 patients had been recruited using a simple random sampling technique. The sample size was calculated using a single population proportion formula in the target population estimated to have adequate health literacy. There is no reasonable estimate since no prior study has been conducted in the Ethiopian setting, so we used 50% (i.e., 0.5) to maximize the sample size [27] and using 95% confidence interval; Zα_/2 =_1.96, the margin of error_=_0.05 and non-response rate=10%.

Before the actual data collection, the list of patients due for follow-up in the NCDs clinic during the 2 months of data collection was obtained from the registration logbook. In the time of data collection, there were aforementioned patients with NCDs in the method section. To select an individual study participant, first the patient’s card was denoted by distinctive code to ease the procedure; then patients’ cards were randomly selected by online random number generator method so that the randomly selected numbers were used to get patients’ card. The cards were used to get patients to collect data in each disease category until the required sample size was obtained.

### Data collection instrument and methods

#### Instruments

CHL was measured by Health Literacy Questionnaire (HLQ) encompassing six of nine HL domains (6). CHL part of HLQ involves 30 questions that cover six multidimensional aspects of CHL domains:

- *Scale 1—Feeling understood and supported by healthcare providers (HPS) (4 items)*
- *Scale 2—Actively managing own health (AMH) (5 items)*
- *Scale 3—Social support for health (SS) (5 items)*
- *Scale 4—Active engagement with healthcare providers (AE) (5 items)*
- *Scale 5—Navigating the healthcare system (NHS) (6 items)*
- *Scale 6—Ability to find good health information (FHI) (5 items)* *[*6*]*

For the domains 1 to 3, participants were asked their level of agreement on HLQ statements and responded as 1 = strongly disagree, 2 = disagree, 3 = agree, and 4 = strongly agree, and for the domains 4 to 6, participants responded as 1 = cannot do, 2 = very difficult, 3 = quite difficult, 4 = quite easy, and 5 = very easy [6]. The overall score for each domain was calculated by adding the item scores and then multiplied by the total number of items in the domain finally divided by the maximum possible score of that domain. The logic behind the use of HLQ is that it measures conceptually distinct scores in each domain that indicate a person’s strengths and weaknesses to their health literacy [6]. As there was normal data distribution, the cut-offs to define “high CHLL” were determined considering those patients who scored mean and above mean from each domain of HLQ items correctly and otherwise as “low CHLL”.

This applied to categorize all the six HLQ domains as there was no standard cut-off [19]*.* In addition to HLQ, instrument includes socio-demographic and socio-economic characteristics (age, marital status, educational status, monthly household income, residence), disease-related factors (presence of co-morbidity, complication/s from NCDs, follow-up visit last 4 weeks), and health information sources. The English version of HLQ, socio-demographic and economic profiles, disease-related factors, and health information sources was translated to Afaan Oromo and Amharic version by experts who were fluent in both languages and translated back to English to check its consistency. Then Afaan Oromo and Amharic version of HLQ, socio-demographic and economic profiles, disease-related factors, and health information sources were used to collect information from the patients. The Cronbach’s alpha for each was more than 0.8. Data were collected through face-to-face exit interview at chronic illness clinic by trained and experienced two nurses who hold Bachelor of Science (BSC) degree using paper-based administered by the nurses pretested translated version of instrument that took less than 20 min to complete.

### Statistical analyses

The statistical analyses were executed by using IBM Statistical Package for Social Sciences (SPSS V23). Before the actual data analysis, data were explored for its completeness, outliers, missing values, and finally cleaned. Respondents’ characteristics were summarized as frequency and percentage. We assessed normality of the data using Shapiro-Wilk test. Multicollinearity test was checked prior to the data analysis and collinearity statistics showed that independent variables were uncorrelated. Given that VIF<10 and tolerance test is less than one for all independent variables. All variables were entered into bivariate logistic regression and independent variables with p-value 0.25 were considered as candidates for multivariable and were analyzed using Backward LR logistic regression analysis. In a multivariable model, variables with p-value less than 0.05 were regarded as statistically significant predictors of CHL in the final model. Hosmer-Lemeshow show goodness of fit test was executed indicating that the model adequately described the data with significance value greater than 0.05.

## Results

### Sample characteristics

A total of 408 participants gave their response giving a response rate of 96.7%. The study participants vary with socioeconomic and demographic backgrounds, chronic diseases’ profile, and disease-related factors (Table 1).

**Table 1:** Sample characteristics in JMC, Ethiopia (*n*=408)

Variables	Category	Frequency (*n*=408)	Percent
Age	18–44	178	43.6
	45–64	117	28.7
	≥65 years	113	27.7
Marital status	Single	57	14.0
	Married	301	73.8
	Divorced	24	5.9
	Widowed	26	6.4
Educational status	Illiterate	153	37.5
	Grade 1–8	124	30.4
	Grades 9–12	75	18.4
	College and above	56	13.7
Monthly household income (1 USD=43.63 ETB)	<10.72 USD	84	20.6
	10.72-21.45 USD	73	17.9
	>21.45 USD	251	61.5
Residence	Rural	189	46.3
	Urban	219	53.7
Chronic diseases profile	Cardiovascular diseases	100	24.5
	COPD	10	2.5
	Diabetes mellitus	96	23.5
	Hypertension	98	24
	Epilepsy	90	22.1
	Asthma	14	3.4
Presence of co-morbidity	One chronic disease	341	83.6
	Two or more chronic diseases	67	16.4
Complication/s from NCDs	Yes	299	73.3
	No	109	26.7
Follow-up visit last 4 weeks	Yes	151	37
	No	257	63

### Health-related information sources

**Fig. 1:**
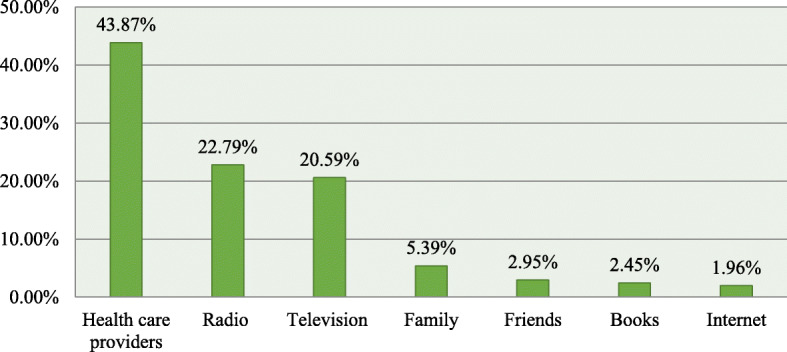
Health related information sources

Forty-four out of 100 study subjects obtain health information from health professions followed by radio which is less than one-quarter 93 (22.79%) of participants using this as a source of information (Fig. 1).

### Communicative health literacy level (CHLL)

Table 2 summarizes that CHLL varies from domain to domain. The study respondents with NCDs possessed high CHLL in four of six (health care provider support, social support for health, active engagement with healthcare providers, and navigating the healthcare system) CHL domains. Participants with NCDs had low CHLL for actively managing health and ability to find good health information (Table 2).

**Table 2:** Communicative health literacy level

HL domains	Mean (SD±)	CHLL	
		High HLL	Low HLL
1. Health care provider support	1.84 (.81)	229 (56.1%)	179 (43.9%)
2. Actively managing health	3.25 (.98)	202 (49.5%)	206 (50.5%)
3. Social support for health	3.97 (1.0)	219 (53.7%)	189 (46.3%)
4. Active engagement with healthcare providers	3.83 (1.11)	229 (56.1%)	179 (43.9%)
5. Navigating the healthcare system	3.37(1.14)	218 (53.4%)	190 (46.6%)
6. Ability to find good health information	2.72 (1.17)	187 (45.8%)	221 (54.2%)

### Factors associated with CHL domain

Table 3 presents multivariable analysis of factors associated with CHL domains. Educational status was significantly associated with all CHL domains except feeling understood and supported by healthcare providers. Whereas, the number of information sources were found to be associated with four of six CHL domains. Study respondents with household monthly income more than 21.45 USD are 4.2 folds and 10.72–21.45 USD are 1.5 times more likely to have high CHLL for actively managing health while female study participants were 50% less likely to have high CHLL for actively managing health. Respondents who reside in urban areas are 3.9 times more likely to navigate the healthcare system. Those participants with NCD who had history of complication/s from NCDs are 0.31 times less likely to find good health information (Table 3).

**Table 3:** Multivariable logistic regression for factors associated with CHL domains

	CHL domain						
		Scale-6 FHI	Scale-5 NHS	Scale-4 AE	Scale-3 SS	Scale-2 AMH	Scale-1 HPS
Variables	Category	AOR (95% CI)					
Educational status	Illiterate	1	1	1	1	1	1
	Grades 1–8	2.34 (1.35, 4.05)*	1.45 (1.09, 2.43)**	1.4 (1.13, 1.65)**	1.14 (1.05, 1.85)*	1.69 (1, 2.85)*	0.74(0.42, 1.3)
	Grades 9–12	2.68 (1.40, 5.14)*	2.28 (1.13, 4.28)**	1.94 (1.03, 3.65)**	2.12 (1.06, 4.25)**	2.32(1.26, 4.27)**	0.82 (0.4, 1.69)
	College and above	5.84 (5.05, 7.89)*	3.23 (1.47, 7.09)*	1.75 (1.47, 3.63)**	2.97 (1.58, 5.58)**	3.87 (1.78, 8.45)**	1.01 (0.44, 2.34)
Number of information sources	One information source	1	1	1	1	1	1
	≥2 information sources	1.67 (1.0, 2.75)**	2.8 (1.75, 4.48)*	0.97 (0.58, 1.62)	1.42 (0.89, 2.28)	1.97 (1.22, 3.18)**	4.2 (2.68, 6.62)**
Monthly household income (1 USD=43.63 ETB)	<10.72 USD	1	1	1	1	1	1
	10.72–21.45 USD	1.57 (1.27, 2.25)**	1.17 (0.56, 2.36)	0.9 (0.46, 1.77)	0.99 (0.51, 1.9)	1.47 (1.23, 5.96)**	.82 (0.41, 1.64)
	>21.45 USD	1.78 (1.09, 3.23)**	2.55 (1.42, 4.59)	0.44 (0.25, 1.77)	0.62 (0.35, 1.1)	4.16 (1.66, 6.03)**	0.78 (0.43, 1.4)
Complication/s from NCDs	No	1	1	1	1	1	1
	yes	0.69 (0.36,0. 92)**	1.05 (0.64, 1.72)	1.25 (0.77, 2)	0.97 (0.6, 1.55)	1.2 (0.74, 1.98)	1.1 (0.67, 1.8)
Residence	Rural	1	1	1	1	1	1
	Urban	0.64 (0.38, 1.1)	0.91 (0.54, 1.54)	3.45 (2.28, 5.19)*	1.60 (1.03, 2.48)**	1.23 (0.75, 2.03)	.676 (.43, 1.05)
Gender	Male	1	1	1	1	1	1
	Female	0.57 (0.36, 0.9)	0.70 (0.44, 1.11)	1.2 (0.78, 1.91)	1.1 (0.7, 1.7)	0.5 (0.32, 0.77)*	0.86 (0.55, 1.33)
Follow-up past 4 weeks	No	1	1	1	1	1	1
	Yes	0.96 (0.59, 1.55)	1.37 (0.86, 2.18)	1.41 (0.91, 2.2)	1.2 (0.79, 1.87)	0.94 (0.6, 1.5)	2.62 (1.40, 2.95)*

NB: *HPS*, healthcare provider support; *AMH*, actively managing health; *SS*, social support; *AE*, active engagement; *NHS*, navigating healthcare system; *FHI*, finding health information; *USD*, United States Dollar; *ETB*, Ethiopian Birr

*Statistically significant association at *p*-value<0.01

**Statistically significant association at *p*-value<0.05

1—Reference category

## Discussion

Health literacy plays central role for the prevention and management of chronic conditions. Indeed, NCDs poses a significant and long-term challenge for patients, health services providers, and the countries health care system. There is a scarce data on CHL among the NCD patients from the JMC, in Ethiopia. To the best of our knowledge, we are the first to assess communicative health literacy and associated factors among adult patients with NCD on follow-up at JMC, Ethiopia. In the present study, patients with NCDs had high CHLL 56.1% (95%CI 52, 60.8) for HL domain health care provider support, 53.7% (95%CI 49.1, 58.3) for social support for health, 56.1% (95%CI 51.2, 60.5) for active engagement with healthcare provider, and 53.4% (95% CI 48.5, 58.3) for navigating the health care system. This indicates a significant number of patients with NCD have a close relationship with at least one healthcare provider who recognizes them well and who they trust can provide useful guidance and information and help them make shared decisions about their health and feel they are supported by health care providers [6]. This provides directions into an individual’s collaboration with the health system and may offer guidance on responsiveness of JMC in addressing individual health literacy needs [28]. A person’s social system plays a key role in that it brings him/her with all the support that s/he wants or needs for health [6]; this implies the health literacy skills and needs of an individual [28]. Patients with NCDs take the initiatives about their health and feel in control in interactions along with healthcare professionals, being able to look for information from other healthcare providers when needed, and they go ahead until they get what they want [6]; they have ability to search about services and assistance; thus, they have all their needs met and promote on their own behalf at the system and service level [6]; this offers awareness into both patients with NCDs and organizational health literacy strengths [28].

Despite the fact it is difficult to compare and contrast as there are no similar studies, the findings are in line with results from other earlier related studies [18, 19] which indicate that CHLL differs domain to domain and country to country irrespective of study population which is to mean cross cutting. Certain demographic and other variables are significantly associated with CHL and will be discussed subsequently.

The present study identified that higher educational status was found to be associated with actively managing health, social support for health, active engagement with healthcare providers, navigating the healthcare system, and ability to find good health information. Study subjects with increased education level are able to have sufficient information to manage their health and improved judgment of assessing health-related information [29]. Individuals with increased educational level are proactive and confident in communicating with health care providers to have information about their health; with the information, they make a sound decision in navigating the health care system which eventually improved HL utmost improved health outcomes [3, 23, 24, 30–32]. On the top of this, having no education hinders them of getting better jobs, having a good salary, and consequently restricts their timely access to health care services which in turn may lead to poor health actions required for managing conditions [19].

Urban residence was significantly associated with social support for health and active engagement with healthcare providers. This implies that urban residents are more likely to have better HL than rural residents [29]. Hence, people residing in urban areas often may have more contact with healthcare professionals and opportunity to be exposed to health promotion programs such as mass, and social media in comparison with the people living in rural areas resulting high CHLL which in turn enhances treatment adherence, effective communication healthcare providers, and ability to manage chronic disease utmost improved health outcome and better self-management for life time [29]. Moreover, it may also be because economic development levels and health resource allocations are lower in rural areas than in urban areas [29, 30].

In the current study, monthly household income was found to be significantly linked with three of six CHL domains: actively managing health, navigating the healthcare system, and ability to find good health information. This indicates patients with high socioeconomic status are able to pay for medication and able to afford to own smart phones which enable to keep abreast of health-related information. Additionally, they can afford to access high-quality healthcare services [20, 24, 33].

This study found that gender was significantly associated with having sufficient information and actively managing health. This means females with NCDs are at risk of having many gaps in their knowledge and lack information on health-related aspects and may neglect their own health [6]. It could be that females in our society are overloaded with multiple responsibilities because of which they may overlook their health. Moreover, socio-cultural issues linked to lower male involvement and support of women’s and lower educational status of women’s is associated with increased perceived barriers of health care access in Ethiopia [34].

The current study found that the numbers of information sources were found to be significantly associated with healthcare provider support, having sufficient information, actively managing own health, navigating healthcare system, and ability to find good health information HL domains. This indicates a significant number of patients with NCDs who obtained information from diverse sources are able to prevent adverse health outcomes such as re-hospitalization resulting from symptom aggravation [19]. This means that clients can obtain reliable and sufficient information from different sources with varying degree and accuracy [22, 24].

The odds in favor of health care provider support for patients’ follow-up visit in the last 4 weeks were 2.62 folds more likely as compared with patients who had not had a follow-up visit in the last 4 weeks. This might be because patients with NCDs attending regular follow-up can establish a good relationship with at least one healthcare provider who knows them well and who they trust to provide useful advice and information and to assist them for shared decision-making about their health and treatment compliance [6]; utmost improved CHL eventually prevents comorbidities and diseases’ complication. A previous study [3] has cautioned that simple follow-up is not a guarantee of improved health literacy unless during follow-up time health care providers give close and holistic follow-up care.

The odds in favor of ability to find good health information for respondents who had complication from disease were 31% less likely to have high CHLL compared with those who had no complications. This implies that significant numbers of patients with NCD feel that there are many gaps in their knowledge and that they do not have the information they need to live with and manage their health concerns [6]. This is in contrast to study [18] stating participants without chronic diseases/without disease complication have low health literacy level. This means an individual without diseases/disease complication does not know what to do compared with the individual with the diseases/disease complication [18]; this could indicate that better HL skills may come from a longer experience [35] in actively using a diverse range of sources to find information and is up to date [6].

This study has many strengths and limitations. It is a novel Ethiopia-based study that assessed robust multi-dimensional HL aspects of adult patients with NCDs and associated factors using HLQ that has previously not yet been available in research or public health setting.

The data quality control was highly practiced throughout data collection as well as data entry and questionnaires were translated into two local languages by experts and pre-tested. The major limitations of this study are as follows: first, this is the first use of the tool in Ethiopia and external validity could be questioned question; second, sociodemographic and economic characteristics of patients are self-reported which imposes to bias; and on the top this, this study is limited to single-centered facility-based cross-sectional only focused on chronic follow-up patients which is difficult to infer for general population.

## Conclusion

Concluding the study, the present study found that patients with NCDs had more than half of high CHLL in four of six CHL domains whereas low CHLL in the rest of two of six CHL domains. The findings of the current study indicate that health literacy levels vary according to socio-demographic and disease characteristics of patients. The score for each individual scale provided detailed and action-oriented picture (insight into key areas in which patients with NCDs can be assisted to have, comprehend, and practice health information to promote and maintain good health) of health literacy competencies and limitations among patients with NCDs. Thus, healthcare professionals should tailor their information and support to the health literacy skills and personal context of their patients, like, encouraging patients to possess high CHLL in the HL domains and give due attention to HL domains with low CHLL. The teach-back method should also be used and enable patients to have high health literacy so that patients can manage their chronic health condition for a lifetime.

### Implication of the study

The current study used HLQ which was designed to measure both innate ability and individual’s self-reported experience of interacting with health care organizations which reflected the health literacy skills and needs of an individual. Hence, the current study pinpoints areas of strengths and challenges of patients’ health literacy with long-term health conditions which is a good insight and highly relevant for JMCor practitioners seeking to intervene. Insights and practical guidance will enable JMC to reform health care service models and implement health literacy principles into routine clinical care that may help with decreasing health disparities and to improve health outcomes of patients with chronic health conditions.

## Data Availability

Due to no consent from the study participants to disclose raw data, this data could not be made available in order to protect the participants’ identity.

## Abbreviations

NCDs: Non-communicable diseases
SPSS: Statistical Package for the Social Sciences
WHO: World Health Organization
COR: Crude odds ratios
AOR: Adjusted odds ratio
HL: Health literacy
CHLL: Communicative health literacy level
JMC: Jimma Medical Center
CVD: Cardiovascular disease
COPD: Chronic obstructive pulmonary disease
DM: Diabetes mellitus
HTN: Hypertension
HLQ: Health Literacy Questionnaire
VIF: Variance inflation factor
LR: Likelihood ratio
SD: Standard deviation
LMIC: Low- and middle-income countries
